# Childlessness and vulnerability of older people in China

**DOI:** 10.1093/ageing/afx137

**Published:** 2017-10-31

**Authors:** Zhixin Feng

**Affiliations:** Centre for Research on Ageing and ESRC Centre for Population Change, School of Social Sciences, Faculty of Social, Human and Mathematical Sciences, University of Southampton, Southampton, UK

**Keywords:** childless older people, Instrumental Activities of Daily Living (IADLs), self-rated health, depression, China

## Abstract

**Background:**

the number of childless older people is increasing in China, but relatively little is known about the role of childlessness in health outcomes. This study investigates the relationship between childlessness and three health outcomes: difficulty with Instrumental Activities of Daily Living (IADLs), self-rated health and depression.

**Methods:**

this study includes 13,171 individuals aged 50 and above from national survey data of the second wave of the China Family Panel Study (2012). Binary/multinomial logistic and ordinary least squares regression models are presented.

**Results:**

childless individuals whose children have all died exhibit worse health outcomes than individuals with children, but this effect is influenced by demographic characteristics, socio-economic status and social security. On the other hand, individuals who are childless due to other reasons (involuntary or voluntary) are less likely to report difficulty with IADLs and to report depression than older people with all children alive after controlling for demographic and socio-economic and social security factors.

**Conclusions:**

the death of a child has an adverse effect on people’s health for both childless people whose children have all died and those who have lost a child but have other children alive. These two groups are in the most vulnerable position, which could also suggest that their children have died because they grew up in a vulnerable family. The government needs to improve the social security for these two groups and provide social services (particularly mental health services) to older people who have lost a child; these could contribute to alleviating some of the adverse effects of the death of a child.

## Background

Along with an increasing ageing population, the number of childless older people in China has also increased. It was estimated that there were 3.52 million childless older people in 2005 [1]. In addition, >30 years of the One-Child Policy has also led to a new ‘childless’ older people phenomenon. Over 1 million families lost their only child in 2010 [2], and this is expected to reach 10 million by 2035 [3].

Three reasons for childlessness in old age have been identified: involuntary childlessness (i.e. biological infertility, mate selection, remaining unmarried or delayed marriage), voluntary childlessness (i.e. conscious decision for a no-parity status due to social attitudes, higher labour marker participation and educational attainment of women) and those which result from the death of one or more children [4, 5]. Childlessness may affect a person’s health status through different mechanisms. From a sociological and economic perspective, involuntary and voluntary childlessness could have positive effects on peoples’ health status if individuals have enjoyed a higher personal consumption (i.e. healthcare) and less pressure to earn an income due to not devoting a significant portion of their income to support children, and avoiding stressors or health issues associated with parenting [6]. Childless persons may also have more assets and a higher disposable income, which allows them to afford better health care and health insurance, and can more easily pay for household services in old age than their counterparts with children [7, 8]. From an intergenerational support perspective, children can provide social support to older parents; they can monitor their parents’ health, and this could result in older parents having better health than their childless counterparts [8]. In addition, childless older people have been found to have worse health behaviours than parents [8]. On the other hand, the death of a child is a traumatic event which could have a long-term effect on older people [9], particularly for those whose children have all died, as they have to cope with health problems by themselves and may have to endure psychological suffering [10, 11]. Therefore, physical and mental health may be different among older people according to different pathways into childlessness.

In traditional China, having no children is generally believed to be an ‘undesirable’ status [5]. It is considered to be a personal tragedy to die without a surviving child in China due to the belief that life after death holds no comfort if one remains heirless [5, 12]. In addition, older people who have no living children could lose their main social support in later life. Although childless older people could receive some institutional support in China, the state welfare provision for older people, including those without children, cannot cover all those in need [5, 13]. Even though China has achieved nearly universal coverage in social security (pension and medical insurance) for older people, inequity among different types of insurances still exists and the formal benefits which older persons actually receive are minimal [14, 15]. Combined with having no social support from children, experiencing inequality within the social security system, witnessing a traumatic event and psychological suffering from losing a child, and being marginalised and sometimes ignored by society, childless people who have lost their child could hold a more vulnerable position in society. The aims of this study are to explore the association between childlessness and health outcomes, and whether childless older people in China are in a more vulnerable position than older people with children.

## Data and methods

### Data

This study uses the 2012 China Family Panel Study (CFPS), which is a national multistage probability sample of Chinese families. There were 35,719 respondents aged 16 and above within 13,281 households [16]. People aged 50 and above were selected for this study. The final sample includes a total of 13,171 respondents for the analysis (there were a further 829 respondents who did not answer all the questions on depression, who were excluded from the analysis).

### Health outcomes measures

Three health outcomes were considered: difficulty with Instrumental Activities of Daily Living (IADLs), self-rated health (SRH) and depression, representing both physical and mental health outcomes. Difficulty with IADLs is commonly used to gauge older people’s daily performance and includes seven items: outdoor activities, eating, kitchen activities, taking public transportation, shopping, cleaning and doing laundry. A binary variable was constructed for IADLs, with 1 representing difficulty with any of the seven IADLs, and 0 representing ‘no’ difficulty with any of the seven IADLs. SRH is a subjective measure of health which has been found to be a sensitive and reliable indicator of current health status for older people [17]. There are three categories: ‘Good’ (excellent, very good and good), ‘Fair’ and ‘Poor’ (unhealthy). In terms of the report of depression, this study uses 20 questions of the Centre for Epidemiological Studies Depression Scale (see Appendix A available in *Age and Ageing* online) to measure how often the respondents felt depressed in the last month which could reflect their psychological distress. For each question, respondents rated the frequency of each symptom of distress using a scale from 1 to 4 (1 = almost never, 2 = sometimes, 3 = often, 4 = most of the time). The scale of depression was constructed by summing up the unweighted numbers in response to 20 questions.

### Childlessness situation

Childlessness is the key variable. The CFPS questionnaire allows respondents to provide information (i.e. birthday, current age, gender, whether he/she was alive in 2012) about up to 10 children. If the respondents provided a valid current age and reported that a child is ‘alive’, this child was treated as being alive in 2012; the number of all living children was then summarised for each respondent, ranging from 1 to 10. Among this group, the analysis further separated those who reported that at least one child had ‘died’. Therefore, there are two groups in the analysis: those with ‘all children alive’ and those who had living children but ‘had lost at least one child’. For those who have no living children, the paper distinguished between those whose ‘child(ren) have all died’ and those who were childless for other (involuntary or voluntary) reasons (‘other childlessness’). The distribution across the four types of childlessness is presented in Table 1. There were 9,039 people with all their child(ren) still alive, 228 people whose children have all died, 3,611 older people who were classified as other childlessness and 296 people who have lost a child but still have at least one living child.

**Table 1.** Descriptive statistics for health outcomes of the whole sample, and those with different childlessness statuses^a^

	Whole sample	All children alive	Children have all died	Other childlessness	People have lost a child but still have at least one living child
	(*n* = 13,171)	(*n* = 9,036)	(*n* = 228)	(*n* = 3,611)	(*n* = 296)
Difficulty with IADLs					
No	87.5%	88.2%	77.2%	87.4%	73.3%
Yes	12.5%	11.8%	22.8%	12.6%	26.7%
SRH					
Good	45.9%	47.0%	36.8%	44.5%	35.5%
Fair	23.0%	22.9%	21.9%	23.7%	17.2%
Poor	31.2%	30.2%	41.2%	31.7%	47.3%
Depression	*n* = 12,342	*n* = 8,451	*n* = 198	*n* = 3,423	*n* = 270
	Range 20–74; mean = 34	Range 20–74; mean = 34	Range 21–67; mean = 36	Range 20–72; mean = 33	Range 21–68; mean = 37

^a^Across four types of childlessness statuses, the *P*-values for three health outcomes are <0.05.

Sources: CFPS 2012, author’s calculations.

### Covariates

Although the focus of this research is on the effects of childlessness on health, it is important to control for other factors so that the effects are reliable. Demographic, socio-economic and social security characteristics are used, including age, gender, urban/rural residence, education, total personal income, marital status, access to different types of old-age insurance and access to different types of medical insurance as control variables following previous reports (i.e. Zhang and Liu [5]).

### Methods

Considering the number of response categories in the three health outcomes, a binomial logistic regression is used to examine the effect of childlessness on difficulty with IADLs; a multinomial logistic regression examines the effect of childlessness on SRH; and an ordinary least squares (OLS) model examines the effect of childlessness on depression. The first model (Model 1) includes only the respondents’ childlessness status in order to explore its direct effect on health outcomes, while Model 2 adds the demographic, socio-economic and social security variables to investigate whether the effects of childlessness on health outcomes are mediated by these factors.

## Results

### Descriptive findings

Table 1 presents descriptive statistics for health outcomes by different childlessness statuses. Most of the older people reported no difficulty with IADLs (87.5%), and around half reported good SRH. The mean score of depression was 34. Older people whose children have all died and those who have lost a child but still have at least one living child are more likely than their counterparts with all their children alive or people who are childless due to other reasons to report difficulty with IADLs, poor SRH (Pearson chi-squared <0.01) and a high score of depression.

In terms of demographic, socio-economic and social security characteristics, key differentials are evident among different statuses of childlessness (Table 2). Older people whose children have all died and those who have lost a child but still have at least one living child are in the most vulnerable position, as they are older (mean age = 71 and 69, respectively), live in rural areas (75% and 80%, respectively), have a lower level of education (68% and 69%, respectively, are illiterate/semi-literate), are less likely to have an income (70% and 80%, respectively) and are less likely to receive a pension after retirement age, compared with older people with all children alive and those who are childless for other reasons. Although people who are childless for other reasons are less likely to have a pension after retirement and medical insurance than parents with all their children alive, they are generally in a better position: they are more likely to have a formal education (55%) and income (44%) than older people with all their children alive (41 and 64%, respectively).

**Table 2.** Descriptive characteristic of the whole sample, and those with different childlessness situations^a^

	Whole sample	All children alive	Children have all died	Other childlessness	People have lost a child but still have at least one living child
	(*n* = 13,171)	(*n* = 228)	(*n* = 228)	(*n* = 3,611)	(*n* = 296)
Age	50–99, mean = 62	50–99, mean = 60	50–92, mean = 71	50–93, mean = 65	50–93, mean = 69
Gender					
Male	49.6%	49.2%	43.9%	51.3%	43.9%
Female	50.4%	50.8%	56.1%	48.7%	56.1%
Residence					
Urban	30.7%	29.0%	24.6%	36.3%	20.3%
Rural	69.3%	71.1%	75.4%	63.7%	79.7%
Education					
Illiterate/semi-literate	48.8%	48.4%	67.5%	46.9%	69.3%
Primary school	18.8%	17.6%	22.8%	21.6%	17.2%
Junior and above	32.4%	34.0%	9.7%	31.4%	13.5%
Income					
None	61.6%	63.0%	70.2%	56.2%	80.4%
Lowest quartile	9.7%	10.3%	8.8%	8.7%	5.7%
Second quartile	10.1%	10.0%	6.1%	11.0%	5.7%
Third quartile	9.0%	8.1%	7.5%	11.6%	5.1%
Highest quartile	9.6%	8.7%	7.5%	12.5%	3.0%
Marital status					
Never married	0.9%	0.1%	0.0%	2.9%	0.3%
Married	84.3%	84.7%	73.3%	85.9%	62.5%
Co-habiting	0.2%	0.2%	0.0%	0.3%	0.0%
Divorced	1.2%	0.9%	0.9%	2.1%	0.0%
Widowed	13.3%	14.0%	25.9%	8.8%	37.2%
Old age insurance					
No pension, after retirement age	24.0%	20.2%	34.2%	32.1%	30.1%
Rural pension	21.5%	18.9%	34.7%	25.8%	39.2%
Urban/rural resident social pension	1.9%	1.9%	4.8%	1.9%	3.0%
Urban resident pension	3.0%	2.2%	4.8%	5.0%	2.4%
No pension, under retirement age	31.4%	38.3%	5.7%	17.4%	11.5%
No pension, still working after retirement age	18.2%	18.5%	15.8%	17.8%	13.9%
Medical insurance					
None	11.0%	10.8%	13.6%	11.5%	9.5%
Public medical insurance	4.7%	4.0%	4.4%	6.7%	3.0%
Urban employee basic medical insurance	11.9%	10.4%	9.7%	16.0%	6.4%
Urban resident basic medical insurance	7.4%	6.6%	9.2%	9.4%	5.7%
Supplementary medical insurance	0.3%	0.3%	0.0%	0.3%	0.0%
NRCMI insurance	64.7%	67.8%	63.2%	56.1%	75.3%

^a^Across four types of children situation, the *P*-values for all characteristics are <0.05.

Sources: CFPS 2012, authors’ calculations.

### Statistical model

The results from the logistic regression models for reporting difficulty with IADLs (Table 3) show that people who had lost children/a child (both childless persons whose children have all died and those who still have at least one child) are significantly more likely to report difficulty with IADLs than their counterparts with all their children alive; while no significant differences are found for other childless persons and older persons with children (Model 1). However, the adverse effect of losing children/a child on difficulty with IADLs among those whose children have all died and those who still have at least one living child become non-significant after controlling for demographic, socio-economic and social security factors. People who are older, female, living in urban areas, illiterate/semi-literate, with no income and never married are more likely to report difficulty with IADLs (Model 2). Only older people who are still in employment after retirement age and without a pension are less likely to report difficulty with IADLs than those under retirement age and without a pension. Other childless persons are less likely to report difficulty with IADLs than people with all their children alive.

**Table 3.** Odds ratios of reporting help needed for performing IADLs, multinomial regression estimates for reporting fair or poor self-rated health and linear regression for depression (the full version of this table is available at *Age and Ageing* online).

	Difficulty with IADLs		Fair/poor SRH				Depression	
	Model 1	Model 2	Model 1		Model 2		Model 1	Model 2
			Fair	Poor	Fair	Poor		
	ORs (95% CI)	ORs (95% CI)	ORs (95% CI)	ORs (95% CI)	ORs (95% CI)	ORs (95% CI)	Beta (95% CI)	Beta (95% CI)
Constant	–	–	–	–	–	–	34.04 (33.85–34.22)***	32.52 (30.78–34.25)***
Childlessness situation								
All children alive (ref:)								
Children have all died	2.21 (1.61–3.03)***	0.92 (0.66–1.3)	1.22 (0.86–1.74)	1.74 (1.29–2.35)***	0.98 (0.68–1.41)	1.23 (0.90–1.68)	1.55 (0.33–2.78)**	0.76 (–0.41–1.93)
Other childlessness	1.08 (0.96–1.21)	0.86 (0.76–0.98)**	1.09 (0.99–1.21)*	1.11 (1.01–1.21)**	0.96 (0.87–1.07)	1.05 (0.95–1.16)	–0.90 (–1.25 to –0.56)***	–0.54 (–0.89 to –0.2)***
People have lost a child but still have at least one living child	2.72 (2.09–3.55)***	1.13 (0.85–1.52)	1.00 (0.71–1.40)	2.08 (1.61–2.69)***	0.86 (0.61–1.21)	1.46 (1.12–1.91)***	2.76 (1.71–3.81)***	1.50 (0.48–2.51)***
Pseudo *R*^2^/adjusted *R*^2^	0.0066	0.1415	0.0019		0.0363		0.0051	0.1034

***P < 0.01, **P < 0.05, *P < 0.1.

Model 2 adds age, gender, urban/rural residence, education, income, marital status, old age insurance and medical insurance.

Sources: CFPS 2012, author’s calculations.

In terms of the results of reporting fair or poor SRH in the multinomial regression models in Table 3, compared with people with all children alive, those in the other childlessness situations are more likely to report poor SRH (Model 1). Again, after controlling for the covariates, the effect of childlessness on SRH becomes non-significant, but those who have lost a child (but have other living children) are still more likely to report poor SRH than people with all their children alive (Model 2). People who are older, female, living in rural areas, with lower formal education than junior level education, lower income and never married/divorced are more likely to report fair and poor SRH than those who are younger, male, who live in urban areas, with junior level education, higher income and are married. Compared with older people under retirement age and without a pension, older people who receive any type of pension or no pension after retirement are more likely to report fair SRH; in addition, those without a pension after retirement or who receive the urban resident pension are more likely to report poor SRH. Compared with those with public medical insurance, people with all the other types of medical insurance are more likely to report fair SRH.

Finally, losing children/a child is associated with a higher depression score for childless persons whose children have all died or those who still have at least one living child than those with all children alive (Model 1 in Table 3). In Model 2, the adverse effect of losing children/a child for those whose children have all died becomes non-significant, but those who still have at least one living child are still more likely to report a higher depression score than those with all children alive after controlling for other factors. Being female, illiterate/semi-literate, never married, divorced or widowed, and without a pension after retirement are positively associated with higher depression; while being childless for other reasons, living in urban areas, having a higher income and receiving a rural or urban resident pension are negatively associated with high depression.

## Discussion

This study investigated the health status of childless older people in China. Without considering demographic, socio-economic and social security factors, childless persons whose children have all died and those who have lost a child but still have at least one living child are more likely to report difficulty with IADLs, poor SRH and higher depression than older people with all children alive; while other childless persons are less likely to report difficulty with IADLs and to report depression, but they are more likely to report fair and poor SRH than parents with all their children alive. The findings partly reinforce previous research showing that childless persons generally tend to have relatively poorer health [8, 18, 19]. After controlling for demographic, socio-economic and social security factors, the findings show that there are no significant differences in the three health outcomes between childless persons whose children have all died and older persons with children. However, people who have lost a child but still have at least one living child are still more likely to report poor SRH and higher depression. This indicates that the death of a child has an adverse effect on people’s health outcomes, both for childless persons whose children have all died and for those who still have at least one living child. Alternatively, both childless persons whose children have all died and those who still have at least one living child are potentially in a more vulnerable position (a lower level of education and less likely to have an income and to receive social security). From a life-course perspective, this may also reveal that their children have died because they grew up in a vulnerable family, and, as a consequence, these older people have increased vulnerability and report poorer health outcomes in later life. It is not surprising to find that other childless persons are significantly less likely to report difficulty with IADLs and to report depression than those with children after controlling for other factors, as the accumulation of positive effects of their early life (i.e. higher personal consumption and less pressure to earn income to raise children) could also result in better health in later life [8].

This study also has its limitations. First, the survey lacks information to separate persons who are childless involuntarily or voluntarily. The health outcomes of these two groups could be different as the motivations to be childless are different. Secondly, due to the small sample size, the analysis was also unable to separate childless persons who have lost their only child from those who have lost all their children. Thirdly, the cross-sectional nature of the study may not capture the short-term effect of losing a child on health outcomes. Nevertheless, the findings herein provide additional insight into the effects of childlessness on health outcomes in China [5, 18].

In conclusion, the findings illustrate that the death of a child has an adverse effect on people’s health both for childless people whose children have all died and for those who have lost at least one child, but have other living children. These two groups are the most vulnerable groups among the respondents (they are older, have a lower level of education and lack income and social security). The findings may also suggest that the children of these groups have died because they grew up in a vulnerable family, with the accumulation of negative effects of vulnerability, and the loss of a child may have contributed to worse health outcomes compared with people with all their children alive. In addition, this group of childless people did not expect to find themselves in this situation, nor were they prepared to receive social support from their children, and, as a result, they may be likely to require greater income support, health care and social services compared with parents [6]. These findings therefore have important policy implications: the government needs to improve the social security for childless persons whose children have all died and for those who have lost at least one child but have other living children. In addition, the common characteristic across these two groups is the experience of losing a child and, in this sense, providing effective social services (particularly mental health services) could contribute to alleviating some of the adverse effects of the death of a child in later life.

**Key points**

- The worse health outcomes are found in childless older persons whose children have all died compared with those with all their children alive.
- Older people who were childless involuntarily or voluntarily are less likely to report difficulty with Instrumental Activities of Daily Living (IADLs), or depression.
- Those who have lost one child but have other living children have poorer health outcomes than parents with all their children alive.

## Supplementary data

Supplementary data mentioned in the text are available to subscribers in *Age and Ageing* online.

## Conflict of interest

None.

## Funding

This research was supported by the AGEGlobe Network funded under the ESRC Ageing and Well-being in a Globalising World (grant number ES/K005979/1) and the ESRC Centre for Population Change (grant number ES/K007394/1).

## Supplementary Material

aa-17-0235-file003
