# Supplementary material for: Childlessness and vulnerability of older people in China
Source: Age Ageing. 2017 Oct 31;47(2):275–81. doi: 10.1093/ageing/afx137 (PMC6016684; doi:10.1093/ageing/afx137)
Supplement: aa-17-0235-file003 [file aa-17-0235-file003.docx]

**Table 3 Odds ratios of reporting help need for performing IADLs, Multinomial regression estimates for reporting fair or poor self-rated health and linear regression for depression**

|  | Difficulty with IADLs | | Fair/ Poor Self-Rated Health | | | | Depression | |
| --- | --- | --- | --- | --- | --- | --- | --- | --- |
|  | Model 1 | Model 2 | Model 1 | | Model 2 | | Model 1 | Model 2 |
|  |  |  | Fair | Poor | Fair | Poor |  |  |
|  | ORs (95%CI) | ORs (95%CI) | ORs (95%CI) | ORs (95%CI) | ORs (95%CI) | ORs (95%CI) | Beta (95%CI) | Beta (95%CI) |
| **Constant** | - | - | - | - | - | - | 34.04(33.85-34.22) *** | 32.52(30.78-34.25) *** |
| **Childlessness situation** |  | | | | | | | |
| All children alive (ref:) |  |  |  |  |  |  |  |  |
| Children have all died | 2.21(1.61-3.03) *** | 0.92(0.66-1.3) | 1.22(0.86-1.74) | 1.74(1.29-2.35) *** | 0.98(0.68-1.41) | 1.23(0.90-1.68) | 1.55(0.33-2.78) ** | 0.76(-0.41-1.93) |
| Other childlessness | 1.08(0.96-1.21) | 0.86(0.76-0.98) ** | 1.09(0.99-1.21) * | 1.11(1.01-1.21) ** | 0.96(0.87-1.07) | 1.05(0.95-1.16) | -0.90(-1.25—0.56) *** | -0.54(-0.89- -0.2) *** |
| Parent who has lost at least a child | 2.72(2.09-3.55) *** | 1.13(0.85-1.52) | 1.00(0.71-1.40) | 2.08(1.61-2.69) *** | 0.86(0.61-1.21) | 1.46(1.12-1.91) *** | 2.76(1.71-3.81) *** | 1.50(0.48-2.51) *** |
| **Age** |  | 1.09(1.08-1.1) *** |  |  | 1.00(0.99-1.01) | 1.01(1.00-1.02) *** |  | 0.00(-0.03-0.03) |
| **Female (ref: Male)** |  | 1.34(1.18-1.52) *** |  |  | 1.14(1.03-1.27) ** | 1.44(1.31-1.58) *** |  | 1.43(1.09-1.77) *** |
| **Urban (ref: Rural)** |  | 0.67(0.56-0.8) *** |  |  | 1.11(0.98-1.27) | 0.81(0.71-0.92) *** |  | -1.40(-1.85- -0.96) *** |
| **Education** |  | | | | | | | |
| Junior or Above (ref:) |  |  |  |  |  |  |  |  |
| Illiterate/Semi-literate |  | 1.72(1.44-2.04) *** |  |  | 1.21(1.07-1.36) *** | 1.62(1.44-1.82) *** |  | 2.30(1.90-2.70) *** |
| Primary school |  | 1.09(0.89-1.34) |  |  | 1.37(1.20-1.56) *** | 1.33(1.17-1.52) *** |  | 0.62(0.18-1.07) *** |
| **Income** |  | | | | | | | |
| No income (ref:) |  |  |  |  |  |  |  |  |
| Lowest quartile |  | 0.61(0.48-0.78) *** |  |  | 1.02(0.87-1.19) | 0.74(0.64-0.85) *** |  | -0.05(-0.57-0.46) |
| Second quartile |  | 0.63(0.49-0.81) *** |  |  | 1.03(0.88-1.21) | 0.56(0.47-0.66) *** |  | -1.71(-2.26- -1.16) *** |
| Third quartile |  | 0.57(0.43-0.76) *** |  |  | 1.20(1.01-1.43) ** | 0.56(0.46-0.67) *** |  | -2.28(-2.9- -1.66) *** |
| Highest quartile |  | 0.7(0.53-0.94) ** |  |  | 1.01(0.84-1.20) | 0.45(0.37-0.55) *** |  | -2.81(-3.44- -2.17) *** |
| **Marital status** |  | | | | | | | |
| Married (ref:) |  |  |  |  |  |  |  |  |
| Never married |  | 1.39(0.81-2.38) |  |  | 1.35(0.82-2.23) | 1.64(1.06-2.52) ** |  | 5.92(4.34-7.51) *** |
| Cohabitation |  | 0.44(0.06-3.31) |  |  | 0.43(0.12-1.51) | 1.67(0.74-3.79) |  | 0.70(-2.53- 3.94) |
| Divorced |  | 1.44(0.82-2.52) |  |  | 1.20(0.81-1.78) | 1.72(1.19-2.50) *** |  | 3.81(2.47-5.15) *** |
| Widowed |  | 1.05(0.91-1.23) |  |  | 1.08(0.93-1.25) | 1.03(0.90-1.18) |  | 2.08(1.59-2.57) *** |
| **Old age insurance** |  | | | | | | | |
| No pension, under retirement age (ref:) |  |  |  |  |  |  |  |  |
| No any pension, after retirement age |  | 1.08(0.86-1.35) |  |  | 1.43(1.21-1.69) *** | 1.50(1.28-1.76) *** |  | 0.48(-0.08-1.05) * |
| Receive Rural Pension |  | 0.77(0.62-0.96) ** |  |  | 1.28(1.08-1.51) *** | 1.12(0.96-1.31) |  | -0.96(-1.52- -0.4) *** |
| Receive Urban-Rural Resident Social Pension |  | 0.72(0.48-1.08) |  |  | 1.65(1.18-2.30) *** | 1.23(0.89-1.70) |  | 0.77(-0.37-1.91) |
| Receive Urban Resident Pension |  | 1.14(0.78-1.66) |  |  | 1.59(1.21-2.10) *** | 1.37(1.02-1.84) ** |  | -1.15(-2.11- -0.19) ** |
| No pension, still in employment after retirement age |  | 0.5(0.4-0.63) *** |  |  | 1.09(0.93-1.27) | 1.00(0.87-1.16) |  | 0.20(-0.3-0.71) |
| **Medical Insurance** |  | | | | | | | |
| Public medical insurance (ref: ) |  |  |  |  |  |  |  |  |
| No |  | 0.98(0.69-1.41) |  |  | 1.42(1.11-1.82) *** | 1.16(0.89-1.51) |  | 0.53(-0.32-1.38) |
| UEBMI |  | 1.15(0.82-1.62) |  |  | 1.36(1.08-1.72) *** | 1.24(0.97-1.60) * |  | -0.19(-0.98-0.6) |
| URBMI |  | 1.09(0.74-1.59) |  |  | 1.90(1.47-2.46) *** | 1.35(1.02-1.79) ** |  | -0.26(-1.15-0.63) |
| Supplementary medical insurance |  | 1.40(0.45-4.34) |  |  | 0.97(0.4-2.38) | 1.01(0.43-2.35) |  | -0.01(-2.91-2.89) |
| NRCMI |  | 1.00(0.7-1.42) |  |  | 1.27(0.99-1.62) * | 1.14(0.89-1.48) |  | 0.47(-0.36-1.30) |
| **Pseudo R2/Adjusted R2** | 0.0066 | 0.1415 | 0.0019 | | 0.0363 | | 0.0051 | 0.1034 |

***p<0.01, **p<0.05, *p<0.1

UEBMI: Urban Employee Basic Medical Insurance; URBMI: Urban Employee Basic Medical Insurance; NRCMI: New Rural Cooperative Medical Insurance.

Sources: CFPS 2012, author’s calculations.

Appendix A

20 questions of the Centre for Epidemiological Studies Depression Scale

| I was bothered by things that usually don’t bother me. |
| --- |
| I did not feel like eating; my appetite was poor. |
| I felt that I could not shake off the blues even with help from my family or friend |
| I felt that I was just as good as other people. (reversed) |
| I had trouble keeping my mind on what I was doing. |
| I felt depressed. |
| I felt that everything I did was an effort. |
| I felt hopeful about the future. (reversed) |
| I thought my life had been a failure. |
| I felt fearful. |
| My sleep was restless. |
| I was happy. (reversed) |
| I talked less than usual. |
| I felt lonely. |
| People were unfriendly. |
| I enjoyed life. (reversed) |
| I had crying spells. |
| I felt sad. |
| I felt that people dislike me. |
| I could not get going. |
